# Elaborating the Role of Natural Products-Induced Autophagy in Cancer Treatment: Achievements and Artifacts in the State of the Art

**DOI:** 10.1155/2015/934207

**Published:** 2015-03-03

**Authors:** Ning Wang, Yibin Feng

**Affiliations:** ^1^School of Chinese Medicine, The University of Hong Kong, 10 Sassoon Road, Pokfulam, Hong Kong; ^2^The University of Hong Kong-Shenzhen Institute of Research and Innovation (HKU-SIRI), Shenzhen 518057, China

## Abstract

Autophagy is a homeostatic process that is highly conserved across different types of mammalian cells. Autophagy is able to relieve tumor cell from nutrient and oxidative stress during the rapid expansion of cancer. Excessive and sustained autophagy may lead to cell death and tumor shrinkage. It was shown in literature that many anticancer natural compounds and extracts could initiate autophagy in tumor cells. As summarized in this review, the tumor suppressive action of natural products-induced autophagy may lead to cell senescence, provoke apoptosis-independent cell death, and complement apoptotic cell death by robust or target-specific mechanisms. In some cases, natural products-induced autophagy could protect tumor cells from apoptotic death. Technical variations in detecting autophagy affect data quality, and study focus should be made on elaborating the role of autophagy in deciding cell fate. In vivo study monitoring of autophagy in cancer treatment is expected to be the future direction. The clinical-relevant action of autophagy-inducing natural products should be highlighted in future study. As natural products are an important resource in discovery of lead compound of anticancer drug, study on the role of autophagy in tumor suppressive effect of natural products continues to be necessary and emerging.

## 1. Introduction

Accumulating studies have revealed the role of autophagy as an important cellular homeostatic process. Autophagy, derived from Greek meaning self-eating, is a self-degradative approach to clear intracellular organelles and proteins [1]. It was first coined by Deter and de Duve in 1960s, with observation that subcellular organelles like mitochondria could be degraded in lysosome of perfused rat liver [2]. Physiologically, autophagy is highly conserved across types of mammalian cells and works in housekeeping manner to scavenge misfolded proteins and damaged organelles, as well as infections [3]. This process is critical for energy balance and genome stability of cells. Autophagy could recycle the nonessential long-term proteins to generate energy in response to nutrient deficiency in starved cells [4]. In general speaking, autophagy is a machinery of cell survival that serves to conquer different types of cellular stress, which may subsequently result in cell death. However, studies in recent years rediscovered autophagy as a mechanism of nonapoptotic cell death, as sustained autophagy may excessively degenerate intracellular structures, and cells undergoing uncontrolled autophagy eventually vanish [5]. It is a morphological definition and no conclusive evidence of specific mechanisms underlying autophagy-induced cell death could be observed [6]. Aberrancy or deficiency of autophagy was observed in various kinds of human disorders such as cardiomyopathy, diabetes, neurodegeneration, autoimmune diseases, infections, liver diseases, and cancers.

It was particularly found that the role of autophagy in human cancer is complicated. On one hand, autophagy may be critical in removing stress induced by infection, hypoxia, nutrient deprivation, and metabolic damage. Autophagy-deficient cells are much susceptible to stress and damage to cell genome, leading to easier tumorigenesis in vivo. It was found that chemical carcinogen-induced hepatocarcinoma could be suppressed by enhanced autophagy which removed aggresome and damaged organelles that could lead to DNA double strand break and genome instability [7], while mice with Beclin-1 knockout are tumor prone [8]. Overexpression of Beclin-1 in mice could develop excessive autophagy to prevent tumor development [9]. On the other hand, activation of autophagy in tumor cells is found to promote tumor development. As tumor cells expand so quickly that nutrients and oxygen could not be sufficiently supplied, tumor cells may undergo serious starvation that causes cell stress. Autophagy in tumor cells is therefore carried out to maintain cell survival by complementing energy supply to conquer nutrient deficiency, and it could remove the damaged organelles and proteins under hypoxia condition [10]. It was observed that inhibition of autophagy led to tumor regression and extended survival of xenografted mice of pancreatic cancer model [11]. However, autophagy may contribute to tumor cell death, in a robust or target-specific way, in cytotoxic agents-treated cancer cells [12]. In recent years, accumulating studies have shown that natural compounds and extracts isolated from medical plants with anticancer property could regulate autophagy in human cancer cells. Phenols, alkaloids, flavones, and organic acids are reported to be autophagy regulators, and it was shown that autophagy may play either cytoprotective or cytotoxic role in natural products-treated cancer cells. With a great interest of finding lead compounds of potential anticancer drug from natural compounds, whose mechanism of action involves autophagy regulation, we summarized the recent advance in studying natural autophagy regulators and discussed the achievements and artifact of the state of the art.

## 2. Natural Products-Induced Autophagy in Cancer

Data was retrieved from publications regarding the regulatory action of natural products on autophagy in cancer cells. Interestingly, almost all the literature refers to the property of natural products in inducing autophagy in cancer. Few studies reported inhibition of autophagy by natural products. Kallifatidis et al. reported that a marine natural compound manzamine A could suppress autophagy in pancreatic cancer cells. Manzamine A could block the fusion of autophagosome and lysosome and abrogate autophagosome turnover [13]. Despite the fact that manzamine A exhibits potent antitumor activity, no direct experimental evidence could show that inhibition of autophagy could contribute to tumor suppression. A recent study revealed that oblongifolin C from* Garcinia yunnanensis* Hu could suppress autophagy and enhance the antitumor effect of nutrient deprivation. As cancer cell develops autophagy as adaptive mechanism towards nutrient deprivation, inhibition of autophagy may lead to death of the cells [14]. However, studies reporting natural products-induced autophagy elaborated more details on the role of autophagy in mediating inhibition of cancer by these compounds (Tables 1 and 2).

### 2.1. Natural Product-Induced Autophagy as a Tumor Suppressor

#### 2.1.1. Autophagy That May Result in Cancer Cell Senescence

Previous studies have revealed that natural products may induce autophagy and cell cycle arrest in cancer cells without significant presentation of cell death. Natural compound isolated from Radix Ophiopogon Japonicus named ophiopogonin B could induce both G0/G1 cell cycle arrest and autophagy in lung cancer cells, without significant apoptosis observed [15]. Sustained arrest of cell phase contributes to cell senescence, during which proliferation rate of cancer cells reduces and motility and invasiveness might be retarded. Pedro and colleagues reported that seven natural prenylated flavones may initiate autophagy in ER positive breast cancer cells while DNA synthesis was suppressed. The proliferation of cancer cells was reduced, though there was no evidence that could directly link up autophagy initiation with proliferation inhibition [16]. Kaushik et al. showed that natural compound honokiol may cause proliferation inhibition which is associated with autophagy and cell cycle arrest [17]. Evidence showing interaction between autophagy and cell cycle arrest is not very clear though some efforts have been made and found that redistribution of cell cycle phase may result from autophagy initiation. Ko et al. showed that autophagy induction may be associated with proliferation inhibition by natural product in cancer cells. In colon cancer cells treated with dimethyl cardamonin isolated from* Syzygium samarangense* (Blume) Merr. & L.M. Perry (Myrtaceae), presence of autophagy inhibitor 3-MA or siRNA against Beclin-1 or Atg5 could restore proliferation of tumor cells [18]. A natural compound penta-1,2,3,4,6-O-galloyl-beta-D-glucose (PGG) may in parallel induce both autophagy and cell cycle arrest. Inhibition of autophagy by the presence of chemical inhibitor or RNA interference in PGG-treated cancer cells results in reentering of cell cycle, suggesting the role of autophagy in mediating cancer cell senescence [19]. However, we cannot simply rule out the possibility that autophagy may be induced due to arrest of cell cycle. Law showed that Alisol B isolated from* Alisma orientale* could initiate cell cycle redistribution and autophagy. Blockade of autophagy in Alisol B-treated cancer cells may result in unfolded protein accumulation-related endoplasmic reticulum stress [20], which indicated that autophagy was initiated to scavenge misfolded protein in Alisol B-treated cancer cells. The accumulation of misfolded protein might be associated with abnormal arrest of cell cycle and aberrant distribution of protein expression profile. In this case, induction of autophagy might be initiated to maintain cell under cell phase arrest conditions by clearing cellular stress. This was further evidenced by the observation that a natural compound Cucurbitacin B could initiate autophagy to conquer nutrient stress induced by DNA damage-associated G2/M cell cycle arrest [21]. As retrieving molecules that could specifically facilitate reentering of cell cycle are hardly obtained, evidence of cell cycle arrest-induced autophagy may not be straightforward and further investigation is still required.

#### 2.1.2. Autophagy as an Exclusive Mechanism of Cell Death

Although many studies have reported that autophagy may be initiated for killing the cells, there is lack of evidence that can be markers and mechanisms of presence of autophagic cell death (ACD). The existence of ACD is therefore under constant criticism, as autophagy is generally observed in dying cells. In this case, autophagy in dying cells may be considered as an automatically raised mechanism for cell survival, even though it is not effective enough to overcome the cellular stress-induced death [5]. Hau et al. reported that coibamide A, a compound isolated from marine Cynabacterium could initiate both apoptosis and autophagy while autophagy may not be essential for cell death or survival, as initiation of autophagy likely occurred in dying cells in response to coibamide A treatment [32]. However, existence of ACD cannot be simply ruled out as many studies have shown that natural products could lead to cell death exclusively with presence of autophagy. Aoki et al. showed that curcumin could initiate autophagy in cancer cells while no apoptosis could be detected, and induction of autophagy is associated with cell death [34]. However, it is contradicting to observe that suppression of autophagy in curcumin-treated cell initiated apoptosis and enhanced cytotoxicity. Autophagy is therefore likely to be a combined mechanism of both cell death and survival. In another study, Meschini and colleagues found that voacamine from* Peschiera fuchsiaefolia* induced tumor cell death dependent on autophagy but not apoptosis [72]. Similar observation was got in other natural products-treated cancer cells including bufalin [28] and triterpenes [68]. The criteria to rule out the presence of apoptosis are pivotal for identifying autophagy as an exclusive mechanism of inducing cell death. Liu and colleagues reported that stellettin A from* Geodia japonica* could induce autophagy as they could observe neither the altered expression of apoptosis marker Bcl-2 nor the appearance of apoptotic nuclei [64]. However, it is not sufficient to conclude with such simple observation on Bcl-2 expression as apoptosis can be initiated by many other pathways. Study from our group introduced a variety of markers to exclude the presence of apoptosis in fangchinoline-treated hepatocellular carcinoma cells, including presentation of Annexin V on dying cells, fragmentation of cell DNA, and cleavage of caspases [40]. Further evidence of nonapoptotic death was obtained to observe that fangchinoline induced cell death was not attenuated by caspase inhibitor. Xie et al. reported in similar way that bufalin-induced colon cancer cell death is independent of caspase activation [27]. As caspases are much exclusively necessary to apoptotic cell death, natural product may have differential mechanisms in inducing cell death in caspase-3-proficient and caspase-3-deficient MCF-7 cells. Rottlerin could induce both apoptosis and autophagy in caspase-3-proficient cells, while in caspase-3-deficient cells, it can only initiate autophagy that was associated with cell death [60]. Furthermore, the exclusiveness of autophagy as mechanism of cell death may be determined in the presence of caspase inhibitor. Wang et al. measured autophagy induced by piperlongumine from* Piper longum* L. in the presence of caspase and necrosis inhibitors. It was found that cell death was attenuated when autophagy inhibitor was added to piperlongumine-treated cancer cells [57]. Observation of this study may confirm that cell death caused by piperlongumine resulted from autophagy induction. Nonetheless, in some cases, apoptosis still could be activated to induce caspases independently [96]. To gain more reliable conclusion, Wong et al. examined autophagic cell death induced by natural compound saikosaponin-D in apoptosis-defective cells [61]. The use of apoptosis-defective cells might be quite self-explanatory but authentication and identification of the cell lines are critical for data interpretation.

#### 2.1.3. Autophagy That Plays a Supportive Role in Apoptosis

As autophagy is usually observed in dying cells, argument was raised to criticize the role of autophagy in promoting cell death, which claimed that autophagy might be an accompanying event that occurs after initiation of apoptosis. Despite the fact that the argument appears to be reasonable since autophagy could be regarded as a mechanism to scavenge dysfunctional organelles and proteins during apoptosis, some studies indeed observed that autophagy may contribute to supporting apoptosis. It was massively considered that autophagy may play a supportive role in inducing cell death though Xavier and colleagues found that, in ursolic acid-induced cell death, apoptosis only contributes to a small proportion while autophagic cell death may be the mass [69]. Saiki et al. showed that, in autophagy-deficient cells, induction of apoptosis by caffeine was attenuated [29]. In most literature apoptosis was found to take the leading role. Xiao et al. found that natural compound curcumin could induce both apoptosis and autophagy, while inhibition of autophagy with small molecule inhibitor 3-MA results in reduced cell death [38]. As a simply obtained autophagy inhibitor, 3-MA was widely used in the studies which would like to distinguish autophagy cell death from apoptosis. Natural compound riccardin D was reported to cause cancer cell death with both apoptosis and autophagy, which could be reduced by presence of 3-MA [97]. A herbal formula Oyaksungisan was found to initiate autophagic cell death while cotreatment of 3-MA rescued the tumor cell [55]. Although these studies may preliminarily try to distinguish cell death induced by autophagy from apoptosis, the use of 3-MA is under criticism as it was found that 3-MA could also block apoptosis. This may lead to overestimation on proportion of cell death induced by autophagy. Another autophagy inhibitor bafilomycin A1 was introduced in some other studies to gain more accurate results. Bafilomycin A1 retards the fusing of autophagosome and lysosome and as a result abrogates autophagy process. Jia et al. used bafilomycin A1 to inhibit autophagy induced by curcumin in leukemia cells and showed that cell death was attenuated in the presence of bafilomycin A1. The results are more compelling but still cannot rule out any nonspecific action of bafilomycin A1, which survives the cells. A better understanding may be obtained if genetic suppression of autophagy-related proteins such as Atg5 could be introduced. Qiu et al. inhibit autophagy both pharmacologically and genetically to show that autophagy contributed to tetrandrine-induced cell death in human cancer [66]. Berberine was shown to induce both autophagy and apoptosis in hepatoma [26], and study from our laboratory measured the proportion that autophagy contributes to cell death induced by berberine. By introducing 3-MA and RNA interference against Atg5, we found that autophagy may contribute to about 10% of the total cell death in berberine's action [25]. This solution seems much reliable, despite the fact that some recent studies also found an autophagy-independent function of Atg5 in triggering death of cell [98]. Knockdown of one more essential protein in autophagy pathway may possibly improve accuracy in technical way. Solution may be also available to examine the time course of autophagy in dying cells. Evodiamine from* Evodia rutaecarpa* could trigger cell death with both autophagy and apoptosis, while the presence of 3-MA reduced the number of dead cells [39]. The induction of apoptosis and autophagy looks parallel in time course, which indicates that autophagy might not just serve as a scavenger after apoptotic cell death is initiated by evodiamine.

The other way by which autophagy may support apoptosis in dying cells with apoptosis is much interesting. Xu et al. examined the relationship between autophagy and apoptosis induced by* Akebia* saponins PA from* Dipsacus asperoides*. When AGS cells were cotreated with autophagy inhibitor bafilomycin A1, the* Akebia* saponins A1-triggered caspase-3-dependent apoptosis was decreased; however, autophagy was not affected in cells with caspase-3 inhibitor [23]. Induction of apoptosis in this case was possibly autophagy-dependent. Similar observation was obtained in bufalin-treated liver cancer cells [99]. Since apoptosis is regarded to be critically controlled but autophagy is generally much more robust process, it is not likely that less fine-tuned autophagy could particularly target proteins that tightly regulate apoptosis pathways. However, recent studies have revealed autophagy may mediate degradation of some particular proteins. This kind of autophagy requires binding of chaperons to help recognition of autophagosome to the targeted protein and therefore was given a term as chaperon-mediated autophagy (CMA) [100]. The mechanism of natural products-induced CMA is not fully addressed; however, study from our laboratory showed that, in timosaponin AIII-induced apoptosis, autophagy may be essential to cause degradation of intracellular inhibitor of apoptosis, XIAP protein. Suppression of XIAP was sufficient to trigger apoptosis in hepatocellular carcinoma. And while autophagy was reduced, apoptosis could not be programmed and timosaponin AIII-treated cells would undergo necrosis instead [67].

### 2.2. A Protective Role of Natural Products-Induced Autophagy on Cancer Cells

The protective effect of autophagy in natural products-induced cancer cell death could be much more straightforward as the process was naturally regarded as cellular stress clearance.  Table 2 listed out compounds that have been reported to initiate a protective autophagy in cancer cells, among which resveratrol is most frequently studied. Resveratrol was found to trigger cytoprotective autophagy in both glioma and melanoma cells [90–92]. Retrieved data reveal that cytoprotective autophagy initiated by natural products was always present with apoptosis. This is not surprising since apoptosis and autophagy may be regulated by some common proteins and signaling. The antiapoptotic Bcl-2 protein is the typical one whose expression may be reduced to trigger apoptosis. Moreover, absence of Bcl-2 could release Beclin-1 to initiate autophagosome formation and as a result both apoptosis and autophagy are induced [101]. The herbal extract Samsoeum (SSE) was shown to trigger both apoptosis and autophagy in cancer cells with reduced expression of Bcl-2 while with an increase in Beclin-1 [62]. Similar observation was obtained in cells treated with methanolic fraction of bitter melon extract [102]. The Akt/mTOR pathway, which is aberrantly activated in cancer, may be another molecular mechanism of crosstalk interconnecting apoptosis and autophagy [103]. While inhibition of Akt triggers cell apoptosis and reduces survival, the subsequent mTOR suppression could initiate autophagy in cells [104]. In this case, natural inhibitors of Akt pathway may be able to induce both apoptosis and autophagy in one cell (Table 2). Data interpretation is much easier as it is not quite difficult to identify the protective role of autophagy technically; however, quantitative measurement on the protection is still problematic, since till now no inhibitor could specifically and efficiently block autophagy in cells. This may lead to either overestimating or underestimating the action of cytoprotective autophagy.

## 3. Discussion

### 3.1. Monitoring a Real Autophagy: A Technical Issue

As a dynamic intracellular process, autophagy is composed of an onset activity, during which autophagosome is formed and fused with lysosome, and an offset activity that facilitates degradation of autophagosome-lysosome complex (also called autolysosome) [105]. Although some major events could be monitored as biomarkers of autophagy, it is far from being accurate to draw any conclusion with only one or two events observed. We are quite sure that the criteria are improving with time; as a result, conclusion from some early literature reporting natural products-induced autophagy in cancer is thought to be too hasty as only least markers were monitored. For example, it was found that flavokawain B from Alpha pricei Hayata could increase LC3-EGFP expression in HCT116 cells [41], but it could possibly happen when lysosome function was simply blocked out, which resulted in increase of both cellular and membrane forms of LC3. This was quite often observed in some early studies of other natural products including honokiol from* Magnolia officinalis* [44], celastrol [30], and tanshinone IIA [65]. As onset of intracellular acidic compartments is a marker of autophagy, Rasul and colleagues monitored magnolol-induced autophagy by staining the acidic vesicular organelle with acridine orange [49]. While increase of acidic particles is usually observed during induction of autophagy, the lysosome protease activity may be regulated independently to autophagy process. In this case, it is still preliminary to draw a conclusion that it was a real autophagy induced in magnolol-treated cells. In some cases, it was found that natural products may initiate the onset of autophagy but block the offset process. Rasul et al. mentioned that matrine may trigger conversion of LC3 to form autophagosome; however, the degradation of autophagosome was impaired as protease activity of lysosome was blocked due to elevated lysosomal pH values in matrine-treated cells [49]. Therefore, conclusion could not be simply drawn by only determining the conversion of LC3. Markers that monitor the offset of autophagy, such as degradation of p62/SQSTM1, should be employed in parallel. The turnover of p62/SQSTM1 is massively operated by lysosome during autophagy; as a result, reduced expression of p62/SQSTM1 may be monitored as a marker of autolysosome degradation [106]. It was found that oblongifolin C from* Garcinia yunnanensis* Hu could induce the number of autophagosomes; however, blockade of lysosome function was found and p62/SQSTM1 turnover was impaired. In this case, whether oblongifolin C could initiate autophagy remains uncertain and requires further investigation [14]. Mei et al. considered autophagy in such a case was aberrant, as study from the research group showed that gambogenic acid could induce accumulation of autophagosome but disrupt p62/SQSTM1 degradation [43]. In addition, natural products-stimulated autophagy should be blocked when pharmacological or genetic inhibitors are present. Practice was made by Lin et al. to monitor baicalin-induced autophagy in bladder cancer cells. Besides autophagy markers were monitored, and cotreatment of 3-MA reduced autophagy initiation by baicalin [24]. Presence of 3-MA helps much in identification of autophagy triggered by natural products, though it could be observed that long-term treatment of 3-MA with natural products such as celstrol controversially increased formation of autophagosome [31]. Genetic inhibition using RNA interference against genes essential for autophagy induction, like Atg5, Atg7, and Atg12, may be required in parallel to confirm a real autophagy is induced. Guidelines for autophagy monitoring and data interpretation are available, which provide comprehensive and restricted methods in evaluating autophagy [107]. It is quite good if all the studies on natural products could strictly follow the guidelines, but the least requirements in identifying autophagy triggered by natural products could be the following: (i) formation of autophagosome: this includes conversion of LC3 from cytoplasmic form to membrane form and increase of autophagic flux; (ii) degradation of autolysosome: it could be illustrated as elevated acidic compartments and turnover of p62/SQSTM1; (iii) induction of autophagy by natural products could be blocked by the presence of pharmacological inhibitors or RNA interference.

### 3.2. Does Origin of Cancer Cells Matter?

As a cellular response mechanism to internal and external stress, autophagy is regarded as robust but highly conserved across different types of cells including tumor cells [108]. The regulation of natural products on autophagy in different cancer cell lines originated from various tumor tissues should be consistent in this regard. Curcumin was reported to initiate autophagy in tumor cells from a variety of cancers including glioma [34], myeloid leukemia [35], glioblastoma [36], uterine leiomyosarcoma [37], and lung carcinoma [38]. It was observed that curcumin could also induce autophagy in colon cancer stem cells, indicating curcumin-provoked autophagy is highly consistent and conserved in nondifferentiated cells as well as well differentiated tumor cells [109]. However, the role of autophagy in determining fate of cancer cells treated with natural products may vary across different types of tumor. It was noticed that bufalin from toad venom is a potent stimulator of autophagy in various types of cancers; nonetheless, bufalin-induced autophagy may play opposite roles in regulating cell death according to results reported by different research groups. It may be due to the technical variations; however, we cannot rule out the influence from genetic variations across different tumor cells, especially when apoptosis is stimulated along with autophagy in natural products-treated cells. Compared with autophagy, apoptosis is under quite restricted control that involves a series of molecules with proapoptotic or antiapoptotic functions. These proteins may be aberrantly expressed due to mutation on cell genome, which results in acquired resistance against acquired apoptosis in cancer cells [110]. Variation of genetic mutations across cancers with different origins leads to various response to natural stimulators of apoptosis. In this case, the role of autophagy in determining cell fate may differ accordingly. The way of apoptosis resistance in influence of the outcome of autophagy may be too complicated to well illustrate currently; however, it was found that, in extreme condition when apoptosis is totally defective, natural products-induced autophagy is mostly contributing to death of tumor cells. It was also noticed that, in noncancerous cells that are sensitive to apoptosis stimulators, acquired autophagy always plays a cytoprotective role to prevent cell death. Taken together, it may indicate the pivot of switch between cytoprotective and cytotoxic role of autophagy may locate at the center of the scale of resistance against apoptosis.

### 3.3. Is Focus on Signaling Pathways That Important?

Retrieved studies have revealed that a single natural product may regulate multiple signaling pathways in cancer cells that could mediate onset of autophagy. Expression of molecules that are directly involved in autophagosome formation, such as Beclin-1, Atg5, Atg7, Atg12, and LC3, was altered upon exposure of the compounds as reported by some studies; however, literature majorly focused on signal transduction through various pathways of kinase like Akt/mTOR, Erk1/2, p38 MAPK, AMPK, and JNK. It was shown that some of these pathways might be responsible for the induction of autophagy. Kim et al. found that blockade of JNK pathway by specific inhibitor SP600125, Samsoeum (SEE), extract-triggered autophagy could be attenuated via suppression on expression of autophagy-related proteins Beclin-1 and LC3 [62]. Nonetheless, in most of studies, it is still difficult to clarify if the pathways are actually responsible for autophagy initiation or just a bypass mechanism that occurs in parallel with autophagy. A herbal extract named KIOM-C was found to activate JNK, which seemed to play a role in generating cellular oxidative and ER stresses in KIOM-C-treated cells [47]. Autophagy was observed in KIOM-C-treated cells; however, it was likely that the initiation of autophagic flux was a spontaneous response to oxidative and ER stress in cancer cells, as there was no evidence to show that autophagy was involved in KIOM-C-induced cell death. Autophagy might be the consequence of stress and was independent of the action of KIOM-C. Neoalbaconol from* Albatrellus confluens* could block the consumption of glucose and ATP generation of cancer cells, as reported by Deng and colleagues, and was able to initiate autophagy. Autophagy is a spontaneous response to energy deprivation in cancer cells [52]. Therefore, signaling transduction induced by natural products is not likely sufficient for autophagy induction, while in cells where autophagy is not responsible for cell death or survival upon natural compounds treatment, autophagy may be regarded as a consequence of changes on other cellular activities induced by natural product treatment [32].

However, study on signaling pathway involved in natural product-induced autophagy may still have significance in elaborating the action of mechanism. For instance, it was found, in different studies, bufalin from Chan-su exhibits both tumor suppressive and cytoprotective autophagy in human cancer cells. The controversial actions of bufalin may be due to the differential effect in inducing changes of signaling pathways. When bufalin was given at lower dose, it may cause endoplasmic reticulum stress, which would initiate autophagy to dispose misfolded proteins, and bufalin-induced autophagy at this dose may elicit protective role to prevent cancer cells from ER stress-induced apoptosis [28, 111]; while the dose goes higher, autophagy may be excessively induced, and cancer cell would undergo autophagic cell death upon treatment of higher dose of bufalin [27, 80]. The differential activation of ER stress-related signaling may mediate switch of the role of autophagy from cytoprotection to cytotoxicity and PERK/eIF2*α*/CHOP was responsible for the interplay between apoptosis and autophagy in bufalin-treated cancer cells [81]. Similar conclusion could be obtained from studies on resveratrol-induced autophagy in cancer cells. Collectively, study on signaling pathway fosters great significance in elaborating the role and action of autophagy. However, as the technical inconsistence was wildly observed, conclusion derived from these investigations may not be convincing enough to illustrate the exact relationship between action of autophagy and the involved signaling pathway. More comprehensive and consistent studies are therefore expected.

### 3.4. Cytotoxic or Cytoprotective? The Cellular Mechanism

Autophagy could be either tumor promoting or tumor suppressive. The tumor suppressive effect may be much more clear, as autophagy may facilitate cell cycle arrest and apoptosis or directly induce cell death. The cellular events in cytoprotective action, in which autophagy helps cancer cell to overcome various cellular stresses, are not clearly elucidated in most of the publications. Recycling of nonessential proteins and organelles to overcome the lack of substrates of metabolism that are required for survival of cancer cells under nutrient deprivation may be possibly one of the major cellular events that fosters the cytoprotective action of autophagy. In some of the studies, researchers observed natural products could suppress mTOR pathway, which was considered as a major mechanism in inducing autophagy. In fact, mTOR signaling mediated translational control of protein synthesis and high activity of mTOR signaling in cancer cells was found in different types of cancer cells. mTOR inhibition is in this case not just the way mediating activation of autophagy but an indicator that cancer cell is trying to overcome nutrient deprivation by restricting nascent protein synthesis. Autophagy induction may subsequently contribute to overcoming nutrient deprivation by future recycling the existing proteins. However, due to technical limit in most of the published studies, direct evidence of natural product-induced autophagy in facilitating recycling of cellular substrates in nutrient-depriving cancer cells is not yet available. Moreover, lack of nutrient and oxygen supply, as well as cytotoxic agent treatment, may lead to damage of proteins and organelles in cancer cells, which brings stress to endoplasmic reticulum and mitochondria. The malfunction of endoplasmic reticulum and mitochondria results in release of proapoptotic factors and causes subsequent cancer cell death. The induction of autophagy by natural products may collectively dispose the damaged proteins and organelles and therefore normalize endoplasmic reticulum and mitochondria function. It was recently shown that blockade of autophagy in bufalin-treated HCC cell could potentiate apoptosis, indicating that bufalin-induced autophagy may be involved in normalizing endoplasmic reticulum [111]. Unfortunately, direct evidence in describing the role of natural product-induced autophagy to stabilize endoplasmic reticulum and mitochondria in cancer cells is not yet available in most of published studies. It is expected in future studies researchers can seek to explore the cellular events occurring in natural product-induced autophagy, which makes elucidation on action of autophagy in a more mechanistic way.

### 3.5. Crosstalk with Apoptosis by Natural Product-Induced Autophagy

As a homeostatic process that responds to cellular stress, autophagy is essential in preventing stress-induced DNA damage and genomic instability [112]. Despite the fact that some previous studies have shown that disruption of autophagy may lead to tumorigenesis, the induction of autophagy in cancer cell may play more complicated roles. While autophagy may counteract the natural product-induced cell death by removing misfolded proteins and damaged organelle to attenuate cellular stress [113], it was also noticed that autophagy initiation may contribute to apoptosis induced by natural products, as evidenced by reduced antitumor action of the compounds when autophagy was blocked. In this case, natural product-induced autophagy is considered a double-edged sword in determining cell fate of human cancers, and interplay between autophagy and apoptosis is recently highlighted in this field of study. Several proteins and signaling pathway may act as scaffold in mediating the crosstalk of autophagy and apoptosis, including p53, Bcl-2 family, DAPK, and JNK [114]. It was particularly noticed that Bcl-2 protein is extensively studied in natural product-induced autophagy and apoptosis. Bcl-2 may interact with both autophagy inducer Beclin-1 and proapoptotic factor Bax to block their function. A series of natural products, such as curcumin, berberine, and evodiamine, was shown to block the interaction between Bcl-2 and beclin-1/Bax and therefore results in autophagy and apoptosis initiation. In addition, activation of JNK pathway was widely observed across various studies that focused on anticancer natural products. As a stress responding pathway, JNK could disrupt the inhibition of Beclin-1 by phosphorylating BIM or Bcl-2. This not only activates beclin-1-dependent autophagy, but also promotes apoptosis by releasing proapoptotic proteins due to the inactivation of Bcl-2. The role of p53 and DAPK in natural product-induced autophagy is not fully revealed, but Wang et al. found that fangchinoline could lead to nuclear translocation of p53, which subsequently activated sestrin2 transcription and initiated autophagic cell death in HCC [40]. These studies revealed that the interplay molecules, which mediate both autophagy and apoptosis activation, may be effective targets in developing cancer killing agents, though the function of autophagy induced still requires critical examination case by case.

## 4. Direction of Future Study

### 4.1. In Vivo Monitoring of Autophagy

The property that natural products could suppress cancers in animal models is quite important for evaluating the druggability of the compounds. More and more autophagy-inducing natural compounds exhibit potent inhibition on tumor growth in vivo. However, although the role of autophagy in determining cancer cell fate could be elaborated on in vitro platform, so far no study could provide compelling evidence showing that in vivo tumor inhibition of natural products involves its regulation on autophagy. This may be due to lack of useful in vivo animal models in which autophagy could be selectively monitored. He and Klionsky reported in vivo monitoring of autophagy in a transgenic GFP-LC3 zebrafish line [115]. Induction of autophagy in zebrafish could be viewed by microscopy in real-time scale. As the approach is simple and noninvasive, the zebrafish model may be quite suitable for high throughput screening of natural products that could initiate autophagy in vivo. However, the model is not disease-related in nature. Studies by Tian et al. developed transgenic mice line expressing GFP-LC3, and the role of autophagy in the pathologic progress of neurological diseases could be monitored by live imaging technology [116, 117]. This successfully established GFP-LC3 transgenic mice which may be applied to elaborate the role of autophagy in carcinogenesis; however, monitor of tumor growth over inner organ may not be available without invading operation. Orthotropic implantation of tumor cells expressing firefly luciferase has been established by our colleagues, to provide a real-time, noninvasive monitoring system on in vivo growth of tumor [118]. Based on this animal model, a dual-bioluminescent reporting system could be taken into consideration in which autophagy as well as tumor growth would be monitored. The Renilla luciferase reporter is the alternative to firefly system and is able to yield reliable results. A previous study has constructed a Renilla luciferase vector-expressing rat LC3 (pRL-rLC3) which demonstrated the possibility and reliability of using pRL-rLC3 to monitor autophagy induction in cell model [119]. Quenching of Renilla luciferin signal would be therefore observed if a treatment could induce autophagy. This hypothesized model may be suitable for monitoring the role of autophagy in the suppression of tumor growth by natural products.

### 4.2. The Synergistic Action of Autophagy-Inducing Natural Products

In recent years, many reviews have summarized the synergistic action of natural product in cancer treatment. Many autophagy-inducing natural products, including berberine, curcumin, and resveratrol, could enhance the therapeutic effect of other agents, and this synergistic effect is correlated with induction of autophagy. Peng et al. found that berberine could enhance tumor-killing action of irradiation by inducing autophagic cell death [120]; curcumin was found to sensitize cancer cells towards treatment of 7-deoxypancratistatin, a novel chemotherapeutic agent [121]; resveratrol-initiated autophagy enhances the cytotoxicity of arsenic oxide on primitive leukemic progenitors, indicating a positive role of autophagy induction in the combination treatment of arsenic oxide and resveratrol against leukemia [122]. The enhancing action of natural products-induced autophagy could also be observed in some herbal extracts including* Koelreuteria henryi* Dummer and* Emblica officinalis* [123, 124]. These studies have shed light on elucidating the mechanistic role of autophagy in mediating the synergistic action of natural products in cancer treatment. In fact, there are still a lot of autophagy-inducing natural products which have been revealed for their capacity of enhancing antitumor action of chemotherapy and radiotherapy; however, the role of autophagy has not yet been fully elucidated. Future study is highly expected to focus on this property of natural products and hopefully the contribution of autophagy in combination therapy could be illustrated in more mechanistic and clinical-relevant approaches.

### 4.3. The Clinical Significance of Autophagy

The ultimate goal of mentioned studies remains to discover autophagy-inducing natural products with significant clinical efficacy in cancer treatment. However, rare published study has focused on clinical trials of autophagy-inducing natural products. In fact, the clinical role of autophagy in cancer treatment is not yet fully elucidated. However, from the data we collected, it is easy to find some compounds may have been used in some countries as anticancer agents. For example, bufalin injection has been used clinically in some parts of China and has shown some therapeutic action in restricting tumor progression in cancer patients. A phase I pilot study showed that bufalin treatment in HCC patients leads to no significant dose-limiting toxicity and improvement of quality of life [125]. Some natural products may exhibit potential of clinical use; for instance, resveratrol, a natural compound universally present in edible and medical plants, has been considered chemopreventive in some previous studies. Phase I clinical trials have been conducted in healthy volunteers, and it was found that consumption of resveratrol did not cause serious adverse effects [126]. However, resveratrol can regulate carcinogen-metabolizing enzymes in human subjects, which can be the mechanism of its chemopreventive action as well as the cause of potential herbal-drug interaction [127]. Clinical study showed that resveratrol alone or in combination with chemotherapeutic agents has beneficial effect on cancer patients [128, 129]. This observation sheds light on the clinical use of resveratrol in treating cancer. Unfortunately, no available information has been disclosed to define the relationship between autophagy and the clinical action of these natural products.

## 5. Conclusion

As a conclusion, recent studies have remarked that autophagy in human cancer cells could be initiated by natural compounds and extracts isolated from anticancer medical plants. Induction of autophagy by natural products may contribute to its tumor suppressive effect by causing cell senescence, inducing apoptosis-independent death, and provoking apoptotic death. Natural products-induced autophagy can also be cytoprotective and cause resistance of cancer cells against death. Higher technical requirement on monitoring autophagy in natural products-treated cancer cells is required to improve study quality in this field. In vivo action of autophagy in mediating tumor regression may be necessary to explore in the future, and significance of synergistic action and clinical relevancy in future studies should be highlighted. Studies in this field will shed light on the development of lead compounds of anticancer drug from autophagy-inducing natural products.

## Figures and Tables

**Table 1:** Natural products that induce tumor suppressing autophagy.

Compound	Plant of origin	Pathway involved	Reference
2-Methoxyestradiol	*Brassica oleracea* var. botrytis	N.A.	[22]
Akebia saponin PA	*Dipsacus asperoides *	JNK, caspase-3 activation↑	[23]
Alisol B	*Alisma orientale *	CaMKK-AMPK-mTOR↑	[20]
Baicalin	*Scutellaria baicalensis* Georgi	Akt↓	[24]
Berberine	Coptidis Rhizoma	Akt/mTOR, CD147↓; Beclin-1, p38 MAPK↑	[25, 26]
Bufalin	Toad venom	ROS, JNK, Atg, Beclin-1, TNF, Atg8↑	[27, 28]
Caffeine	Coffee beans	Erk1/2↑; P70S6K, S6, 4E-BP1, Akt↓	[29]
Celastrol	*Tripterygium wilfordii *	LC3-II, MAPK, Beclin-1↑; Akt, mTOR, S6K, MEK↓	[30, 31]
Coibamide A	Marine cyanobacterium	N.A.	[32]
Crotoxin	South American rattlesnake	N.A.	[33]
Curcumin	*Curcuma longa *	Erk1/2, Beclin-1, LC3-II, AMPK↑; Akt/mTOR/p70S6K↓	[34–38]
Dimethyl cardamonin	*Syzygium samarangense* (Blume) Merr. & L.M. Perry (Myrtaceae)	N.A.	[18]
Evodiamine	*Evodia rutaecarpa *	Bcl-2/Beclin-1↓	[39]
Fangchinoline	Radix Stephaniae tetrandrae	AMPK, sestrin2, p53 translocation↑	[40]
Flavokawain B	Alpha pricei Hayata	N.A.	[41]
Furanodiene	Rhizoma curcumae	N.A.	[42]
Gambogenic acid	Gamboge	Beclin-1↑	[43]
Honokiol	Officinal Magnolia Bark	Beclin-1↑; Akt/mTOR↓	[17, 44]
Indirubin	*Isatis indigotica* Fort.	N.A.	[45]
Jia-Wei-Xiao-Yao-San	N.A.	N.A.	[46]
KIOM-C	N.A.	JNK, ROS, CHOP↑	[47]
Liensinine, isoliensinine, dauricine, cepharanthine	N.A.	AMPK↑; mTOR↓	[48]
Magnolol	Officinal Magnolia Bark	Bax/Bcl-2 ratio↑	[49]
Matrine	Radix Sophorae flavescentis	Endosome/lysosome pH value↑	[50]
Neferine	*Nelumbo nucifera *	ROS↑; GAH, PI3K/Akt/mTOR↓	[51]
Neoalbaconol	*Albatrellus confluens *	PDK1, PI3K, Akt, HK2↓, glucose consumption, ATP↓	[52]
Nexrutine	*Phellodendron amurense *	N.A.	[53]
Oblongifolin C	*Garcinia yunnanensis* Hu	Lysosome cathepsin↓	[14]
Oleifolioside B	*Dendropanax morbifera* Leveille	Atg3, LC3-II↑; Nrf2↓	[54]
Ophiopogonin B	Radix Ophiopogon Japonicus	pAkt/mTOR/p70S6K↓	[15]
Oyaksungisan (OY)	N.A.	JNK↑	[55]
Penta-1,2,3,4,6-O-galloyl-beta-D-glucose (PGG)	N.A.	Unfolded protein response, MAPK8/9/10↑	[19]
Pheophorbide-a	*Scutellaria barbata *	Erk↓	[56]
Piperlongumine	*Piper longum* L.	ROS, p38 MAPK↑	[57]
Plumbagin	*Plumbago zeylanica *	PI3K/Akt/mTOR↓	[58]
Riccardin D	*Dumortiera hirsuta *	Caspases cleavage↑	[59]
Rottlerin	*Mallotus philippinensis *	N.A.	[60]
Saikosaponin-D	N.A.	ER stresses, unfolded protein response, cytosolic calcium, AMPK↑; sarcoplasmic/endoplasmic reticulum Ca(2+) ATPase pump↓	[61]
Samsoeum	N.A.	AMPK, Erk, Beclin-1, LC3-II↑; Akt, mTOR↓	[62]
*Solanum nigrum* leaves extract	N.A.	N.A.	[63]
Stellettin A	*Geodia japonica *	ER stresses↑	[64]
Tanshinone IIA	Salviae miltiorrhizae	Erk↑	[65]
Tetrandrine	Radix Stephaniae tetrandrae	N.A.	[66]
Timosaponin AIII	*Anemarrhena asphodeloides *	AMPK↑; XIAP, mTOR↓	[67]
Triterpenes	*Ganoderma lucidum *	Beclin-1↑; p38 MAPK↓	[68]
Ursolic acid	*Bupleurum falcatum* L. (Umbelliferae)	JNK↑	[69]
Viriditoxin	Jellyfish *Nemopilema nomurai *	LC3-II, Atg5, Atg7, Beclin-1↑	[70]
Vitexin 6	*Byrsonima crassifolia *	N.A.	[71]
Voacamine	*Peschiera fuchsiaefolia *	N.A.	[72]
Weikang Keli	Root of *Codonopsis pilosula*, Rhizoma Atractylodis Macrocephalae, Rhizoma Curcumae Aeruginosae, Rhizoma Pinelliae, *Actinidia chinensis* Planch, and *Rhodiola rosea *	N.A.	[73]

**Table 2:** Natural compounds that induce cytoprotective autophagy.

Compound	Plant of origin	Pathways involved	Reference
*α*-Eleostearic acid	*Momordica #x2009;charantia *	Akt↓, Erk1/2↑	[74]
Anthricin	*Anthriscus #x2009;sylvestris* (L.) Hoffm.	Akt/mTOR↓	[75]
Arenobufagin	Toad venom	PI3K/Akt/mTOR↓	[76]
*β*-Elemene	Zedoary	Atg5↑, Akt/mTOR↓, Erk1/2↓	[77–79]
Bufalin	Toad venom	LC3-II↑, Beclin-1↑, Atg7↑, Atg12↑, AMPK↑, mTOR↓	[80, 81]
Crotoxin	South American rattlesnake	Not applicable (N.A.)	[33]
Cucurbitacin I	*Cucumis #x2009;sativus* L.	Beclin-1↑, Beclin-1/Bcl-2 interaction, HIF-1*α*↓	[82]
Dioscin	Soybean	N.A.	[83]
Englerin A	*Phyllanthus #x2009;engleri *	Akt↓, Erk↓, AMPK↑	[84]
Gossypol	Cotton seeds	Interaction between Bcl-2 and Beclin-1↓	[85]
Isobavachalcone	*Fructus #x2009;psoraleae *	N.A.	[86]
Mollugin	*Rubia #x2009;cordifolia* L.	Erk↑ PI3K/Akt/mTOR↓	[87]
Parthenolide	Feverfew	AMPK↑	[88]
Physalin A	*Physalis #x2009;alkekengi* L. var. *franchetii* (Mast.) Makino	Beclin-1↑	[89]
Resveratrol	Japanese knotweed	Erk1/2↑, p38 MAPK↑, Atg5↑, Beclin-1↑, LC3-II↑, Akt/mTOR↓,	[90–92]
Sesbania grandiflora flowers	N.A.	N.A.	[93]
Wogonin	*Scutellaria #x2009;baicalensis *	mTOR↓, Raf/Erk1/2↓	[94]
Zearalenone	*Fusarium #x2009;graminearum *	LC3-II, Beclin-1	[95]
